# Effectiveness of time-related interventions in children with ADHD aged 9–15 years: a randomized controlled study

**DOI:** 10.1007/s00787-017-1052-5

**Published:** 2017-09-27

**Authors:** Birgitta Wennberg, Gunnel Janeslätt, Anette Kjellberg, Per A. Gustafsson

**Affiliations:** 1 Child and Adolescent Psychiatry, Center for Social and Affective Neuroscience, Department of Clinical and Experimental Medicine, Faculty of Medicine, Linköping University, Linköping, Sweden; 2 Department of Public Health and Caring Sciences, Disability and Habilitation, Uppsala University, Uppsala, Sweden; 3 Centre for Clinical Research Dalarna, Falun, Sweden; 4 Department of Social and Welfare Studies, Faculty of Medicine, Linköping University, Norrkoping, Sweden

**Keywords:** Children, ADHD, Time perception, Time-assistive devices, Intervention

## Abstract

Specific problems with time and timing that affect daily routines, homework, school work, and social relations have been recognized in children with ADHD. The primary treatments for children with ADHD do not specifically focus on time-related difficulties. The aim of this randomized controlled study (RCT) was to investigate how multimodal interventions, consisting of training in time-processing ability (TPA) and compensation with time-assistive devices (TAD), affect TPA and daily time management (DTM) in children with ADHD and time difficulties, compared with only educational intervention. Thirty-eight children on stable medication for ADHD in the 9–15-year age range were randomly allocated to an intervention or a control group. The children’s TPA was measured with a structured assessment (KaTid), and the children’s DTM was rated by a parent questionnaire (Time-Parent scale) and by children’s self-reporting (Time-Self-rating). The intervention consisted of time-skill training and compensation with TAD. Data were analysed for differences in TPA and in DTM between the control and intervention groups in the 24-week follow-up. Children in the intervention group increased their TPA significantly (*p* = 0.019) more compared to the control group. The largest increase was in orientation to time. In addition, the parents in the intervention group rated their children’s DTM as significantly (*p* = 0.01) improved compared with the parents in the control group. According to the children, their DTM was not significantly changed. In conclusion, a multimodal intervention consisting of time-skill training and TAD improved TPA and DTM in children with ADHD aged 9–15 years.

**Electronic supplementary material:**

The online version of this article (doi:10.1007/s00787-017-1052-5) contains supplementary material, which is available to authorized users.

## Introduction

In everyday life, not only adults but also children have to deal with issues concerning time. Daily time management (DTM) is important in becoming independent, autonomous and for performing everyday activities successfully [1, 2]. DTM is how a person can transfer a plan to performance, and accomplish and finish everyday activities in the right order and within a certain time interval. Examples are to do morning chores in good time and to hurry up if needed. The International Classification of Functioning, Disability and Health Children & Youth version (ICF-CY) is composed of the components *Body functions and structures*, *Activities and Participation, Environmental factors,* and *Personal factors.* According to the ICF-CY, there are two codes specifically defining time in daily life: managing one’s time [d2305] and adapting to time demands [d2306] which together can be referred to as DTM. Both codes are defined in the component of *Activities and Participation* (general tasks and demands). DTM is partially based on a person’s mental function of time-processing ability (TPA). TPA develops during childhood and adolescence, starting with time perception, followed by time orientation and time management. Time perception (experience of time and durations of activities) and time orientation (i.e. to tell the time and know which day and month it is) are basic levels of TPA and are necessary for time management [2, 3]. Time management is a superordinate concept to describe planning, ordering events in chronological sequences and allocating the amount of time for activities. Time management develops last and is part of the higher level cognitive functions/executive functions. In the ICF-CY, there are three codes related to TPA: experience of time [b1802], orientation to time [b1140], and time management [b1642]. These codes are defined in the component of *Body functions* (experience of time and time functions, orientation functions and higher level cognitive functions).

Children and adolescents (hereafter referred to as ‘children’, according to the United Nations [4]) with ADHD have been shown to have deficits in executive functioning, including planning [5–7]. They also have problems in organizing materials and activities, time management and planning (OTMP) [8–10]. Specific problems with time and timing have also been recognized in children with ADHD [9–12]. Children with ADHD seem to have a different sense of time than typically developing children [13]. Their ability to discriminate and reproduce time intervals and to make retrospective time estimations has been shown to be impaired [12, 14]. Noreika et al. [11] state in a review that persons with ADHD have timing deficits in motor timing, time estimation and temporal foresight. Only one article was found investigating time-based prospective memory and the relation to time perception in children with ADHD [15]. Time-based prospective memory is of interest as remembering what to do in due time is needed for time planning [16]. Mioni et al. [15] found that children with ADHD performed less precisely on time-based prospective memory tasks and used less efficient clock-checking strategies than typically developed children of the same age. This is in line with earlier research showing that children with ADHD or learning difficulties might have a TPA equivalent to that of younger children [17, 18]. Children with ADHD have been shown to have a delayed maturation of the cerebral prefrontal regions that are important for executive functions, including time management [6, 19]. This could be an explanation for their delayed TPA. The consequences in daily life are difficulties in automatising routines, understanding the concept of time, achieving an overview of time, being on time for an appointment, starting and completing daily activities independently, planning and completing long-term projects, understanding and using a calendar, and hurrying up if required [5, 9, 20]. This difficulty affects all aspects of life: daily routines and homework, school work, and social relations [9, 10]. ADHD is shown to be persistent [9, 10, 21] and children diagnosed with ADHD have been found to have continuing problems with DTM as adults [22]. These problems take the form of procrastination, and missed appointments and deadlines [8, 23].

The primary treatments for children with ADHD are psychosocial treatments and/or pharmacological treatment [10, 24]. In the recommendations from the Swedish National Board of Health [10], assistive technology for cognition (ATC) is recommended as a complement to other interventions for children with ADHD.

Psychosocial treatment is often offered as parent and/or teacher programs. Psycho-education aims to inform about the ADHD diagnosis and its treatment and to give the opportunity to discuss strategies for managing ADHD symptoms in daily life. Psycho-education has been shown to achieve good effects, especially as regards parents’ understanding of their child’s difficulties, and to improve interaction between parents and children [25], but also to reduce ADHD symptoms [26]. Behaviour parent training and behavioural classroom management have been developed for many years, and are well studied and are well-established treatments [27]. Parent training shows significant benefits on parent/teacher ratings of children’s ADHD symptoms [27] and reduces parent’s stress and increases parent confidence [28]. These treatments thus focus primarily on disruptive behaviours.

Psychosocial treatments focusing on training organizational skills and strategies have been developed to target deficits with OTMP in children and adolescents with ADHD [27]. OTMP interventions meet criteria for a well-established treatment [27]. OTMP interventions might be performance oriented, based on the child not having motivation to perform the skill, and the intervention can consist of parents and teachers setting specific individual daily goals for the children, and prompting and monitoring the children to ensure they achieve these goals. The intervention Parents and Teachers Helping Kids Organize (PATHKO) is performance oriented and sessions are primarily focused on parents and teachers. Intervention with PATHKO gained significantly better organizational functioning, compared to a waiting list control group [7]. Psychosocial interventions focusing on OTMP might also be skill based, i.e. based on the child not having the skill, and the intervention, therefore, focuses on helping children to learn new tools and routines [7]. In a review of Evans et al. [27] the Organizational Skills Training (OST) and the Homework, Organization, and Planning System (HOPS) were studied. In the OST intervention, children learn to record assignments and expiration dates, organize school papers into binders, and use checklists. The intervention includes 20 individual sessions for the children and the parents participate in the last minutes of the sessions. Parent and teachers are supposed to prompt, praise and reward skills use. OST is designed for children in elementary school. Intervention with OST produced significantly better parent and teacher ratings of organization, compared to a waiting list control group [27]. In a comparison to PATHKO, the OST intervention was significantly better on organization measures [7]. The HOPS intervention is based on a similar model of training as OST, but for older children (in middle schools). Children train with a coach and in a similar way as in the OST intervention, but the children also learn to plan homework completion. Results indicate significantly better parent ratings for organization, but teacher ratings do not show significant effects [27]. In a study of Abikoff et al. [7] children receiving OTMP interventions in addition to organizational improvements also made significant progress in attention and social skills compared to a control group. In a meta-analysis of organizational interventions for children and adolescents with ADHD, OTMP training leads to moderate improvements as rated by teachers, and large improvements rated by parents [29]. However, all OTMP interventions have mainly focused on organization skills in a school context and in school work, e.g. to organize the material that is necessary in the school situation and to do homework in time [25, 29–31]. We found no study with OTMP interventions in children with ADHD, focused specifically on time management in daily activities outside the school context.

Pharmacological treatment of ADHD reduces the core symptoms of ADHD [24, 32–34] and can hence improve OTMP deficits, but still a considerable number of children and adolescents on medication show continuing difficulties in time and planning and, therefore, need additional treatment [8, 35, 36].

Assistive technology for cognition has been developed within the last 20 years. According to the ICF-CY, ATC is in the component *Environmental factors*. Gillespie et al. [16] state in their review that the most frequent use of ATC is to assist with the executive functions of time management and organization and planning. Time-assistive devices (TAD) are ATCs aimed at compensating for difficulties in time management, orientation to time, and experience of time. Examples are reminders/alarm clocks, weekly schedules or step-by-step-schedules with text or pictures, and timers visualising time graphically as dots or a surface that decrease in numbers or size [16, 37–39]. TAD may not only be high-tech, e.g. apps for smartphones and electronic planning devices, but can also be low-tech, consisting of paper and pictures [10, 16]. Greater independence, a sense of self-control and increased DTM have been reported for children, adolescents and adults with autism, acquired brain injury, developmental disabilities, intellectual disabilities (ID) and psychiatric illness [16, 23, 39–44]. Most studies have focused on adults with acquired brain injury [16]. Nonetheless, the effectiveness of interventions to increase DTM has not yet been sufficiently studied in randomized controlled studies [43–45]. Only two studies were found on TAD and persons with ADHD. Lindstedt and Umb-Carlsson [42] studied how professional support and TAD facilitate everyday life and promote community participation of adults with ADHD. Nineteen patients with ADHD, all on ADHD medication, participated in the study. Most participants had an additional psychiatric diagnosis. The most frequent support was to carry out a daily routine and the most valued TADs were weekly schedules and watches/alarm clocks. In a randomized controlled study, Janeslätt et al. [41] evaluated an intervention with TAD in children with intellectual and developmental disabilities, including children with ADHD. Thirty-eight children (five with ADHD) aged 6–11 years were allocated to intervention or control groups. The intervention group was offered education to parents and school staff, adaptations in school, and TADs. The intervention lasted for 6 months. The results imply that DTM can improve and that the intervention group improved their TPA significantly more than the control group. However, the children in this study were younger than in our study, and only a few of them had been diagnosed with ADHD.

To our knowledge, there have been no multimodal intervention studies focusing on DTM and TPA in children with ADHD aged 9–15 years outside the school context. Therefore, it is of interest to investigate the impact of time-related interventions on difficulties in DTM and TPA in children with ADHD on medication. Our hypothesis was that participants in the intervention group would improve their DTM and their TPA significantly compared to participants in the control group.

### Aim

The aim was to investigate how a multimodal intervention, consisting of training in TPA and compensation with TAD, affects TPA and DTM in children with ADHD aged 9–15 years, compared with only an educational intervention.

## Method

### Study design

This multicentre randomized controlled (RCT) study was conducted on children aged 9–15 years with ADHD in Sweden. It was designed as a randomized study following the CONSORT statements [46] with an intervention group and a control group with different extents of treatment [47]. In this study, the control group was offered only one of the three interventions offered to the intervention group.

Before conducting an RCT study, it is recommended that the design, data collection and intervention should be investigated in a real-world setting to make it possible to make any changes needed [47]. A pilot study was, therefore, conducted with five children with ADHD aged 9–15 years. The purpose of the pilot study was to test if the data collection and the content and length of the intervention were feasible and acceptable for the participants. The results indicated the feasibility, based on parent’s, children’s, coaches’ and occupational therapists’ evaluations.

The intervention was designed to be multimodal, based on guidelines for interventions for children and adolescents with ADHD [10, 24] and had three components: advocacy, compensation, and remediation [48]. Both groups were exposed to advocacy in the form of education for parents and coaches. The education was given for three reasons: first, as a basis for parents and coaches to increase their understanding of deficits in the child’s TPA and how this could affect the child’s DTM; second, to motivate the parents and coaches to facilitate the participation of the child in the intervention; and third, for ethical reasons to offer at least some form of intervention to all children.

Subsequently, the intervention group received the additional two components: compensation and remediation. The compensation component consisted of working around the problem using different types of TAD, finding individual compensation strategies and structuring the physical environment [48], all according to every child’s needs.

In the remediation component, the children were supported in training time skills by a coach. This intervention component was based on work from Langberg et al. [25, 31] with coaches supporting the child to learn organizational skills. Training with a coach was chosen to make it easier for the child to perform the training often, regularly and in an everyday environment at school or at home.

**Fig. 1:**
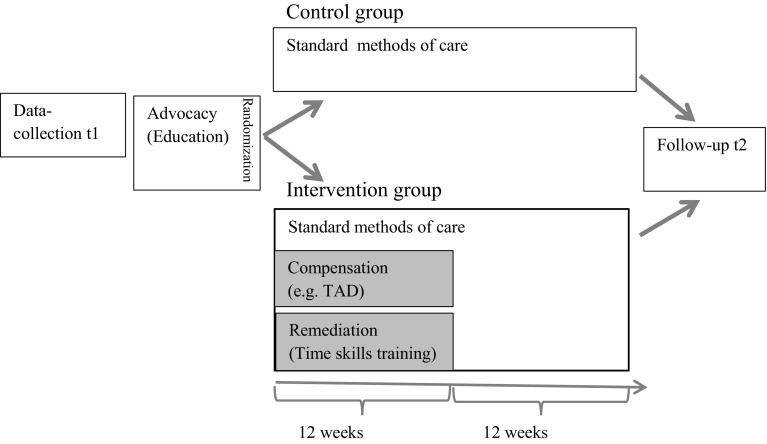
Overview of the design, from inclusion in the study to the follow-up

Data collection was done at baseline (t1), both for the intervention and the control group. The intervention lasted for 12 weeks. The choice of the length of intervention period was based on clinical experience and previous studies training OTMP [7, 20]. Then there was an implementation phase of another 12 weeks, when neither of the groups received any treatment beside standard methods of care, i.e. medication and/or psychological support. After 24 weeks, there was a follow-up assessment (t2). This study design was chosen based on clinical knowledge that starting to use an assistive device takes some time to be integrated with the person’s everyday life [49, 50]. As regards the control group, in addition to the education day, the child and family were only offered standard methods of care (see Fig. 1). After 24 weeks, the children in the control group were offered interventions (data not shown).

### Study population and recruitment

Participants were recruited from three child and adolescent psychiatric clinics (CAPs) and one children’s habilitation service (HAB) in Sweden between September 2012 and March 2015. The clinics represented large cities, small towns and sparsely populated areas. All the children had had a steady prescription of ADHD medication for at least 3 months, and were evaluated by their physician or the first author (BW) for inclusion in the study.

Inclusion criteria were a diagnosis of ADHD, age 9–15 years and parent-reported difficulties with DTM, despite medication for ADHD and ten points or more on a clinical rating of 15 statements related to problems with DTM. The statements were chosen from the subscales “Planning and organizing”, “Time concepts” and “Coping in learning” of the questionnaire “Five to Fifteen” (FTF) [51] made by the physician or the first author (BW) together with the parents. Examples of statements were if the child was stressed by time limits, had difficulties in using a watch or being on time for an appointment, or if the child was “lost in his/her own world” and was not aware of the passage of time. Further examples were when a child had obvious difficulties in carrying out and completing morning chores and arriving in time for school, had difficulties in calculating the time span for daily activities and leisure activities, or forgot booked appointments. Parents judged the child’s problems by rating the statements “not correct” (0p), “sometimes correct” (1p) or “correct” (2p). The exclusion criteria were autism spectrum disorder, ID (IQ < 70), or language barriers (e.g. not being able to answer questionnaires in Swedish).

Parents of the children with ADHD who met the above criteria were asked to participate in the study during normal monitoring visits to the CAPs or HAB. Information about the study’s purpose and structure was provided orally, and in writing in the form of a booklet with brief information. Parents were also asked to enrol a person (preferably a teacher or another member of the school staff) to function as the child’s coach. Parents who agreed to participate received written invitations for a personal visit for the purpose of data collection, one invitation for the parents and an adapted version for the child, accompanied by a consent form.

### Procedure and intervention

Baseline data collection (t1) took place at the outpatient clinic in the course of two visits (in less than one-third of the cases only in one visit). The number of visits was based on the occupational therapist’s knowledge of the child’s ability to cope and travelling time to the outpatient clinic. Written informed consents were collected from both parents (and older children), before or at the start of the first visit. At the first visit information was given to the child and the parents about the study and the randomization process. Data collection was carried out with the child and the parent separately.

During the visit/visits, the parents met a trained occupational therapist for an interview to identify and prioritize everyday issues that restricted or impacted the child’s performance in everyday living, and to respond to the Time-Parent scale questionnaire. They then met with an assistant to respond to demographic questions. The child met a research assistant/the first author to respond to a questionnaire (data are not used in the present study) and the trained occupational therapist for an assessment (KaTid), and to respond to the self-rating questionnaire Time-Self-rating. If needed, the child was offered help by reading or explaining the questions in the self-rating questionnaires.

Interventions were consistently scheduled to occur twice a year. When a group of children (usually four or six in the same local area) with informed consent were enrolled, and baseline data collection were completed, parents and coaches received a (1-day) manualized education session. The last author generated a randomization list matched for age and sex in accordance with random.com and the children were randomized 1:1 for intervention or control groups. Information about assignment to intervention or control groups was given to the parents at the end of the education session (see Fig. 1).

The education lasted for 6 h and was given in groups. Each child’s parents and coach were invited to participate, along with additional school staff if they wanted to. The education included group discussions, exchanging experiences and discussing strategies. The focus was on lectures about DTM and the development of TPA in typically developing children as compared with children with ADHD. It also contained information about the consequences of TPA deficiencies in daily activities and how to find strategies to compensate for these consequences and how to support children with deficiencies in time-related skills. The instruction was manualized and GJ was responsible for the content and conducted all sessions.

The additional intervention for the intervention group (compensation and remediation) lasted for about 12 weeks, with treatment sessions provided by an occupational therapist and a coach supporting the child. The occupational therapist met the child and parent(s) to give feedback about the assessment findings. In this meeting, the occupational therapist, the parent(s) and the child identified and decided on one to three goals, and made an individualised plan for the compensation intervention. The occupational therapist also met the coach and the parents separately to give feedback on the assessment findings and to give instructions for the training.

The first component, compensation, included three or four treatment sessions (lasting for about 1½ h), with both parents and the child participating. The focus was on finding compensating strategies for the child, structuring the physical environment, and prescribing TAD. The intervention was tailored to the individual needs of the child [16, 41, 48]. Examples of compensating strategies could be to establish and maintain functional morning and evening routines and spreading homework over smaller units of time. Examples of structuring the physical environment could be to organize clothes and sportswear so that they were easy to find and to make sure there were clocks visible in all rooms at home. Prescribing of TADs included choosing an adequate TAD and introduction of the TAD, and later follow-up on the use of TAD.

In the second component of the intervention, remediation, in which the child performed time-skill training, the child was supported by a coach. The time-skill training was in the form of “challenging tasks”. The concept of challenging tasks was inspired by “My Time” (in Swedish Min Tid), a program designed for children with ID. In the present study, the challenging tasks were specially developed for children and adolescents with ADHD (by GJ and BW). The challenging tasks consisted of 14 tasks of increasing complexity, starting with collecting experiences of time duration, followed by training in time orientation and later on in time management. An example of a task in time perception was to measure, in minutes, the duration of five self-chosen recurrent activities and document in a binder. Another example was to compare the amount of time needed to perform the same activity in two different ways, e.g. to walk to school or to bike to school. One example of a task training time orientation was to use a computer program to learn to tell the time. In training time management, the child used the self-measured activities and decided how many of them could be fitted into a 45-min period. Another example was to document school activities in a calendar and to check the calendar every day. All the challenging tasks were gathered in the binder, together with instructions on how to perform the tasks. Every child was supposed to complete ten different tasks. Four out of the 14 tasks were exchangeable, depending on the age and maturity of the child. The child was supposed to do the challenging tasks for 20 min/day and was supported by the coach in a short meeting one to three times/week during the training period.

All coaches who supported the child were offered supervision by an occupational therapist at two sessions during the training period, as a group session or an individual session by telephone. At these meetings the challenges and the way to support the child could be discussed.

The occupational therapists responsible for the intervention had experience of working with children with disabilities and time difficulties. All of them were certified KaTid raters before entering the study. KaTid certification includes a 3-day course for professionals, complemented with homework and a number of KaTid assessments of children with and without disabilities. The occupational therapists in the present study also participated in the education day, along with the parents and coaches.

### Treatment integrity

Treatment integrity was controlled for in the following ways. The content of the education day was manualized and also the time-skill training. The coaches and the occupational therapists documented when and what was done in every training session and in meetings with the parents, in a special protocol. During the study, the occupational therapists were invited to attend recurrent days of seminars in groups to discuss interventions and to ensure that the same setup was used. They were also offered individual support by GJ and BW, via telephone or e-mail, if needed.

### Subjects

The ADHD diagnosis was determined in accordance with DSM-IV criteria by an experienced CAP clinician after a thorough neuropsychological investigation encompassing careful clinical examination, and monitored with the help of both questionnaires and, in most cases (> 90%), a computer-based assessment of core symptoms of ADHD: QbTest (Qbtech. Quantitative behaviour technology. https://www.qbtech.com/. Accessed 13 Jan 2017).

The parents of 65 children (46 boys, 19 girls) were asked to participate. The mean age at inclusion was 11.6 years (range 8.6–16.1, SD 1.90). Written informed consent was received for 46 (71%) children (32 boys, 14 girls), and they were randomly allocated to the intervention/control group. Of these, eight (17%) did not complete the follow-up assessment after 24 weeks, or withdrew their consent or were excluded from analysis (in one case) because of administrative problems. This resulted in 19 children each in the intervention and control groups, respectively, who were evaluated. The number of girls was 10 (26%), five in each group. There were no significant differences between the 38 completers and the eight dropouts concerning age, sex, time measures (see below) on intake ratings.

**Fig. 2:**
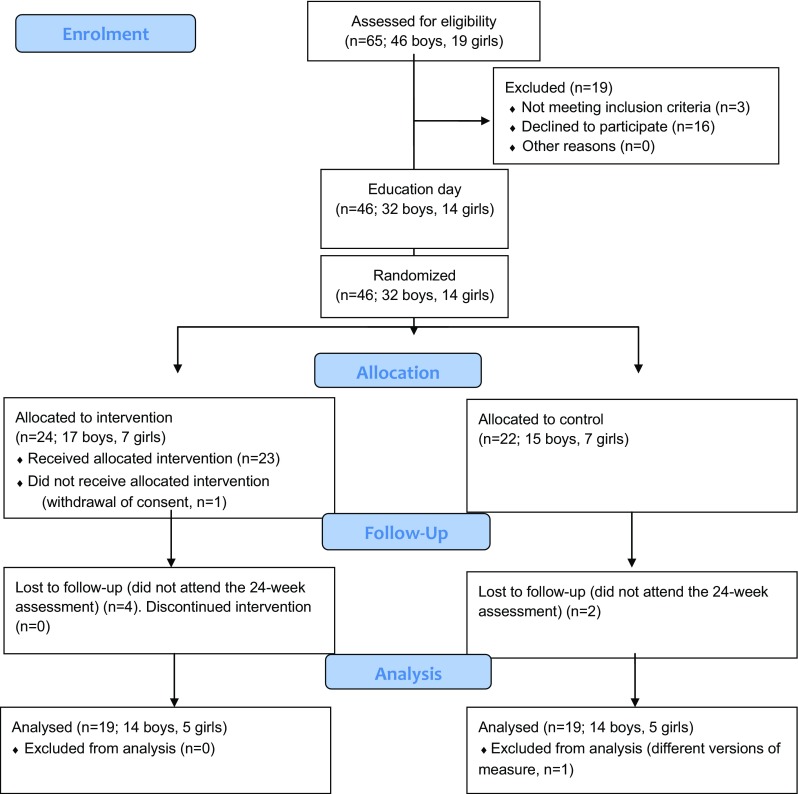
Flow diagram

The flowchart (Fig. 2) presents the allocation of participants, attrition, and remaining participants in the analysis.

### Instruments

The Kit for assessing time-processing ability (KaTid) [3] is an instrument for assessment of time perception, time orientation and time management for children of a developmental age of 5–10 years (KaTid-Child) and adolescents with a developmental age of 10–17 years (KaTid-Youth). Time perception in KaTid is comparable with the ICF-CY:s code b1802, experience of time, including knowing the duration of activities. Time orientation and time management is comparable with the codes b1140 and b1642 in the ICF-CY. The child responds to questions and performs practical exercises, such as setting a timer for a fixed time and placing picture sequences in the correct order. This instrument is interdisciplinary and the testing is performed by a trained professional person, e.g. an occupational therapist. KaTid-Child contains 57 items measuring time perception (15 items), time orientation (32 items) and time management (10 items), summarized into one measure of TPA. KaTid-Youth contains the same sub-groups, but consists of 59 items, 33 of which are the same as in KaTid-Child. KaTid-Child and KaTid-Youth have been shown to have good validity and reliability with children and adolescents, with and without disabilities, in measuring the same construct and measuring change [3, 41]. KaTid-Child has been validated and tested for internal consistency in children with and without disabilities—the Cronbach alpha was 0.78–0.86 [2, 52]. In this study, KaTid-Youth version 19 was used for children from 10 years of age and KaTid-Child version 18b for the youngest children. The same version was used for each child at baseline and at follow-up.

In the Time-Parent scale questionnaire [2], the parents judge their children’s DTM. The questionnaire consists of 12 statements rated on a Likert agreement scale with five response options scored from “do not know” (0p), “never” (1p) to “always” (4p). The Time-Parent scale has been validated and tested for internal consistency, for children and adolescents with and without disabilities, with a Cronbach alpha of 0.79–0.86 [2, 52].

The Time-Self-rating instrument is designed to capture children’s own experience of DTM [53]. It contains 21 statements concerning DTM and 7 items concerning strategies, and has a Likert scale with 4 response alternatives in frequency, scored from “never” (1p) to “always” (4p). The instrument is validated in children aged 10–17 (*n* = 83) with disabilities using Rasch analysis. The results indicate that 21 items of the Time-Self-rating fitted into a unitary construct measuring DTM, reliability was high (0.82) yielding a separation value of 2.15, altogether good psychometric properties [53]. In the present study, the 21 items measuring DTM were employed.

### Statistical analysis

For the KaTid instrument, raw scores were used both as a total score of TPA and for each subscale: time perception, time orientation, and time management.

Sample size was based on findings from an RCT study, [41] including children with disabilities aged 6–11. The intervention group (*n* = 17) had an average increase of 0.9 logits and the control (*n* = 20) increased by 0.35 logits (SD: 0.59) in Time-Parent scale during intervention. A power analysis with an independent *t* test with equal variance showed that a group of 40 children with 80% certainty can discern an increase of 10% at the 5% level.

Demographics were analysed with descriptive statistics. Preliminary analysis with a Shapiro–Wilk’s test [54] confirmed whether the assumptions of normality held for the different variables.

On the basis that there were only two measurement points we used a per-protocol analysis to evaluate the effectiveness of the 12-week intervention, followed by a 12-week implementation period. A change score was calculated for KaTid (sum and on each subscale), for the Time-Parent scale and for the Time-Self-rating. This was done by subtracting the baseline score (t1) for each child from the score at the 24-week follow-up (t2). ANCOVA was used with the difference score as the dependent variable; group, sex and living situation as fixed factors and age as a co-variate. Effect size (ES) was used to analyse the magnitude of the differences in TPA (assessed with KaTid) and in DTM (rated by parents with the Time-Parent scale and by children with the Time-Self-rating) between the control and intervention groups at the 24-week follow-up. It was calculated using Cohen’s *d* with ES *d* = 0.2–0.5 representing a small effect, *d* = 0.5–0.8 medium and *d* = 0.8 large effect [55].

To evaluate if there were any differences between the sample analysed and the sample lost at follow-up a sensitivity analysis was conducted. Missing data were imputed for the KaTid total score, the subscales time perception, time orientation and time management, as well as the Time-Parent scale and the Time-Self-rating scale, assuming that the difference between intake and 24-week follow-up would be 0 for the dropouts in both the intervention and control groups.

The data were analysed using the Statistic Package for Social Sciences (SPSS) version 24.0 (SPSS Inc. Chicago, IL, USA), selecting a significance level of 0.05 and a confidence interval of 95%.

## Results

At baseline, the children in the control group had somewhat higher total scores in TPA in all subscales except for time management, where they had somewhat lower scores. At baseline, the children in the control group had somewhat lower scores for DTM as rated by parents and lower DTM for the children’s self-ratings.

The demographics of the children who participated in the study are presented in Table 1.

**Table 1:** Demographic data and baseline characteristics

	Intervention group (*n* = 19)	Control group (*n* = 19)
Gender *n* (%)		
Girls	5 (26)	5 (26)
Boys	14 (74)	14 (74)
Mean age (SD; min–max)		
	11.7 (1.83; 9.2–15.1)	11.1 (1.71; 8.6–13.5)
Medication *n* (%)		
Long-acting stimulant	14 (73)	17 (89)
Atomoxetine	2 (11)	2 (11)
Long-acting stimulant + atomoxetine	3 (16)	0 (0)
Living with *n* (%)		
Both biological parents	7 (36)	13 (68)
Shared living	5 (26)	1 (5)
One biological parent	2 (11)	2 (11)
One biological parent and a step-parent	3 (16)	2 (11)
Other	2 (11)	1 (5)
Parent’s national origin *n* (%)		
Both Swedish	15 (79)	15 (79)
One Swedish	1 (5)	3 (16)
Neither Swedish	1 (5)	0 (0)
No answer	2 (11)	1 (5)
Parent’s civil status *n* (%)		
Married/domestic partnership	9 (47.5)	13 (68)
Divorced	9 (47.5)	4 (21)
Other or no answer	1 (5)	2 (11)
Parent’s education *n* (%)		
University/college	13 (34)	10 (26)
High school/upper secondary school	8 (21)	11 (29)
Vocational education	13 (34)	12 (32)
Elementary school	1 (3)	3 (8)
No answer or not relevant	3 (8)	2 (5)
Time-processing ability (TPA) mean (SD)		
KaTid—*Sum*	45.3 (9.50)	46.5 (8.84)
KaTid—*Time perception*	9.0 (3.42)	10.2 (3.59)
KaTid—*Time orientation*	28.8 (6.37)	29.2 (5.22)
KaTid—Time management	7.5 (3.31)	7.2 (3.44)
Daily time management (DTM) mean (SD)		
Time-Parent scale	21.1 (2.99)	19.5 (3.36)
Time-Self-rating	55.2 (8.27)	50.5 (10.52)

### Intervention fidelity

At least one parent of all the children in both the intervention and the control groups attended the education day, and also the vast majority of the coaches. All children and their parents in the intervention group attended the stated number of intervention sessions with the occupational therapist.

The number of challenges accomplished varied for each child from three to all ten challenges that were planned (mean: eight challenges). All children received one to four TADs, mostly one or two. The majority of the TADs were for compensating for deficits in time perception or time orientation. An example of a product compensating for deficits in time perception was a visual timer. Examples for time orientation were an electronic day schedule and weekly or annual schedules in plastic-coated paper. A few children (the oldest) received TADs compensating for deficits in time management, a web calendar synchronized to the child’s own mobile phone or a mobile calendar adapted for the child.

### Time-processing ability

Regarding the global score of the TPA, all children increased from baseline to follow-up, but the children in the intervention group increased their TPA significantly more compared to the control group (*p* = 0.019), as measured by the KaTid total score. Regarding the subscale *time perception*, children in the intervention group increased their scores significantly (*p* = 0.046), while the children in the control group declined. Regarding the subscale *time orientation*, all children increased from baseline to follow-up. However, the increase in the intervention group was significantly larger (*p* = 0.010). Regarding the subscale *time management*, both groups increased slightly—children in the intervention group more than children in the control group, but not significantly (Table 2).

**Table 2:** Differences between pre-intervention and follow-up for TPA (time-processing ability) and DTM (daily time management) mean (SD), *p* values, effect size and number of participants (*n*)

	Intervention group (*n* = 19)		Control group (*n* = 19)		*p* value	Effect size
	Pre-intervention	Follow-up	Pre-intervention	Follow-up		
	Mean (SD)	Mean (SD)	Mean (SD)	Mean (SD)		
TPA						
KaTid—*Sum*	45.3 (9.50)	50.4 (7.70)	46.5 (8.84)	48.1 (9.12)	0.019	0.38
KaTid—*Time perception*	9.0 (3.42)	9.8 (3.10)	10.2 (3.59)	9.9 (3.44)	0.046	0.29
KaTid—*Orientation to time*	28.8 (6.37)	32.5 (4.26)	29.2 (5.22)	30.4 (5.81)	0.010	0.42
KaTid—*Time management*	7.5 (3.31)	8.2 (3.30)	7.2 (3.44)	7.7 (3.69)	ns (0.764)	0.03
DTM						
Time—Parent scale	21.1 (2.99)	25.0 (4.48)	19.5 (3.36)	20.3 (5.01)	0.011	1.0
Time—Self-rating	55.2 (8.27)	55.3 (9.18)	50.5 (10.52)	54.1 (11.06)	ns (0.117)	− 0.37

Using Ancova and Cohen’s *d*

### Daily time management

Children in both the intervention and control groups increased their DTM according to parental ratings. However, the parents in the intervention group rated their children’s DTM as significantly more improved than the parents of children in the control group (*p* = 0.011) (Table 2).

The children’s self-ratings for DTM were higher at follow-up in both groups, with no significant differences (Table 2).

## Dropouts

Out of the 46 randomized children, 38 children did the follow-up and were analysed. Regarding the dropouts, one child in the intervention group did not start the intervention at all because of withdrawal of consent. Four children in the intervention group and two children in the control group were lost at follow-up. One child in the control group was excluded from analysis because of the use of different versions of the KaTid instrument at baseline and at follow-up. There were no significant differences between the 38 completers and the eight dropouts concerning age, sex, KaTid total score and subscales, Time-Parent scale or Time-Self- ratings at intake. In the sensitivity analysis missing data were imputed for KaTid sum score, the subscale time perception and time orientation as well as the Time-Parent scale, assuming that the difference between intake and the 24-week follow-up would be 0 for the dropouts. The results for these scales were still significant (*p* < 0.05). See supplementary Table 3.

## Discussion

This study investigated how time-related multimodal interventions can affect TPA and DTM in children with ADHD who have time and planning difficulties.

The remediation component (training of TPA) combined with the compensation component (including prescription of TADs) significantly improved the TPA in time perception and time orientation, compared to only education (the advocacy component) and standard methods of care. The ESs were small. Regarding the most complex TPA ability, time management, there was no significant intervention effect. The ES were smaller than those found by Janeslätt et al. [41] where TPA increased with a strong effect. The latter study included younger children (age 6–11) and a 2-h education session for parents, and the intervention lasted 6 months, while the present study included a full-day education session and a 12-week intervention period with both a compensation and a training component. In Janeslätt et al. [41] the children also had different cognitive problems and only a few of them had ADHD. The larger ES in TPA in Janeslätt et al. [41] might indicate that it was an advantage to introduce TADs at a younger age, but it could also be explained by the longer intervention period, the time of second measurement or the differing diagnoses.

The present study adds detailed results in the three subcategories representing the three levels of TPA. The improvement in the intervention group in comparison with the control group appeared for time perception and time orientation, which are the two lower levels of the TPA and crucial for developing time management. Thus, the intervention mainly increased in the lower levels of TPA. This could be explained partially by the children’s age. Many of the children were of ages where time management is not yet expected to be very developed [57]. It could also be explained by the goals decided: most of the interventions focused on difficulties in time perception (in durations of activities) and time orientation. The setup and content of the challenging tasks also promoted these levels. Based on that, the first challenging tasks focused on time perception and time orientation, which are the lower levels of TPA, and the latter addressed time management. We know that every child coped with the first challenges but the last ones were undertaken by fewer children. This could explain primarily why the lower levels of TPA developed most.

The perception of time is mainly researched in experimental studies [11, 12]. In the present study, the children collected experiences of time by measuring the durations of daily activities in a real-life setting. It is known that it is necessary to know the duration of activities to be able to estimate time, and to estimate time is often difficult for children with ADHD [11]. The fact that time-skill training provides better TPA is new knowledge as regards children with ADHD. The results are in line with the results in a recent study, in which children aged 10–17 years with ID trained in a similar way [58]. The preliminary results in this study showed that the intervention group increased their TPA significantly more than the control group, with a medium ES (Cohens *d* = 0.55). These results, together with the results from the present study, may indicate new options for children with different developmental disabilities to improve TPA by training.

Most of the challenging tasks were focused on everyday activities to make it easier for the children to transfer the experiences to activities in daily life. The training was manualized, and supported by a coach to help the child to perform the training often and regularly. This is in line with OTMP training results, in which children increased their ability to organize schoolwork after training in concrete everyday situations with a coach [25, 31].

Parents in the intervention group rated the DTM of their children to be significantly more improved than parents in the control group. The remediation component and the compensating component including intervention by TAD, presumably lie behind this significant effect on the children’s DTM. The benefit of TAD is consistent with previous studies in which children, adolescents and adults with developmental disabilities, ID, psychiatric illness and acquired brain injury showed greater independence and participation when using TADs [16, 23, 39–44]. However, as we know, this is the first study focusing on TADs for children with ADHD. The TADs considered in this study were mainly used to compensate for deficits in time perception and time orientation, but TADs used to compensate for deficits in time management were also used for the older children. This use of TADs compensating for different levels of TPA is in good agreement with previous research in the field [16, 37–39, 41, 59].

Unlike their parents, half of the children in the intervention group rated their DTM as equal or worse after the intervention, compared to before. Studies of parent’s and children’s estimation of the child’s health-related quality life (HRQOL) show that they do not always match [60] and in studies of children’s self-reported problems, children have tended to report more problems than their parents [61]. The best agreement is shown in domains that can be observed, with less agreement in non-observational domains [62, 63]. Some studies on children with ADHD show agreement between parents and children concerning HRQOL [64, 65] and some studies do not [64, 66]. These mixed patterns can have different explanations: that a child with ADHD is more impulsive, inattentive and hyperactive and maybe answers questionnaires quickly without much reflection [66]. Another explanation could be an effect of response-shift, defined as a person’s view of his or her ability to change over time [67]. The children in the present study may, as a result of self-reporting measurement, participation in determining goals for intervention, and the intervention, have become more aware of their abilities and limitations, and thus rated their skills more realistically after intervention. This could perhaps explain why children in the intervention group that had tested their abilities in the challenges judged their DTM to be only slightly improved, while the children in the control group judged their DTM as much higher at follow-up. In a study examining assistive technology for cognition supporting everyday life for adults with ADHD, the participants experienced less satisfaction with domestic chores and leisure activities after intervention. An explanation given by the authors was increased awareness of the limitations in performance [42]. The same pattern of awareness of limitations in their child’s performance in daily time management could of course also be expected in the parents’ judgement at follow-up. This did not happen. Parents in the intervention group judged their children’s DTM as significantly more increased at follow-up. This could of course be influenced by the fact that they were not blinded for the intervention; moreover, they were part of it. Regardless of whether there is good agreement between the child and the parent or not, it is important to request both perspectives when possible, since they provide complementary and important information about the child’s health and well-being [63–66].

The children in the control group did not increase their TPA or DTM markedly (according to their parents), even though their parents received education that especially focused on time issues. Previous studies of psycho-education have shown good effects on parents’ understanding of the child’s difficulties and improved interaction between parents and children [25]. It is also known that a combination of interventions, usually medication and psycho-education, achieves better results than a single intervention [10, 24]. In a study by Janeslätt et al. [41], an intervention consisting of both education and compensation was highly effective. Our study indicates that education alone is not enough to improve TPA and DTM in children with ADHD. Children who have difficulties with DTM despite medication probably need interventions that more specially target time and planning difficulties, together with training and compensation using TAD.

## Methodological considerations

The intervention lasted for 3 months. After another 3 months, with only standard methods of care, there was a follow-up. Data collection was performed at baseline and at follow-up, not following the last intervention session. It might have been possible to have both a post-intervention test and a follow-up, which would have strengthened the study. This was unfortunately not possible in the present study due to limited access to the target group. When investigating the effect of an intervention, it is often preferable to have more than two measurement times [68]. Ploghart and Vandenberg [68] suggest at least three measurement times. A longer follow-up period with a third data collection would, on the other hand, have resulted in an even longer waiting period for the control group, which would probably have reduced the number of participants remaining in the study.

The multicentre study design offered an opportunity to get participants from a variety of different geographical areas, which is considered as a strength. A weakness could be that several people were active in the data collection and intervention, which required a well-prepared schedule to coordinate how the assessments and the intervention were carried out. To ensure the interventions provided were consistent, seminars were held to discuss and acquire new knowledge and products for occupational therapists involved in intervention. The risk of deviations from the study design arises if the intervention design is more complex, i.e. if interventions are offered by many different persons [69]. Intervention fidelity was, therefore, of special importance in this study. It was checked by considering attendance at the education day and the recurring meetings with the occupational therapist, the quantity of completed “challenging tasks”, and TADs received by the children. An extended way to improve the intervention fidelity could have been an extended education and supervision of the coaches to make sure there was compliance with the remediation component. In addition, video/audio recordings could have been used to monitor how the coaches supported the children.

In the present study, there could of course be some bias because parents, children and those who carried out the measurements knew which children had or had not received the intervention. Trying to reduce this potential bias, the pre-intervention results were not available in the second data collection. Parent ratings could of course also be “affected by expectation bias”, but these types of measures are not inferior to observational data according to Bikic et al. [29]. The occupational therapists that performed the follow-up were also independent of the intervention provided. In addition, both objective instruments (the KaTid), and questionnaires (the Time-Parent scale and the Time-Self-rating) were used.

During the intervention, the children’s active participation in the assessment, in goal-setting, and in the intervention was important. In a review by Wessles et al. [49], important aspects of assistive devices to be used include if the user’s opinions are considered in the selection of a device, if the user is motivated, and if the devices are checked regularly. In the present study, the intervention design with children’s active participation, may have contributed positively to the results. Children with ADHD can have difficulties planning ahead and sticking to the plan [9, 32]. Despite this, the interventions were implemented with significant results in both TPA and DTM and almost all children completed he intervention period. At follow-up, however, 17% dropped out of the study, which is quite high in a study with a relatively short intervention period, but still less than in the study of Janeslätt et al. [41].

The ICF-CY is composed of the components *of Body functions and body structures*, *Activities and Participation*, and *Environmental factors.* Transferring the instruments that were used in the present study to ICF-CY vocabulary shows that they embrace the *Body function* component as well as the *Activity and Participation* component. The KaTid instrument measures a child’s time-processing ability, which can be attributed to the *Body function component*; the Time-Parent scale and The Time-Self-rating instruments measures DTM, which can be attributed to the *Activity and Participation component*. The use of these instruments attributed to two health-related components in ICF/CY could be considered as a strength [70]. However, an even more extensive battery of instruments could have also included the *Environmental component.*

One weakness of this study is the relatively small number of participants and, therefore, caution should be employed in generalizing the results. However, the recruitment was from regular CAP and HAB from different services in Sweden, and the sample should, therefore, mirror a population with children with ADHD suffering from time and planning deficits despite medication. A randomization was also carried out in an effort to avoid systematic bias. According to the relatively small number of participants there is a need for further research with larger groups in children with ADHD. Further research is also needed to investigate the long-term effects of time-related interventions.

## Conclusions

Multimodal time-related intervention using remediation and compensation increased TPA and DTM in children with ADHD aged 9–15 years with time deficits. It could be recommended that TPA and DTM should be measured to identify difficulties in TPA and daily functioning in children with ADHD. The results point at the possibility of offering interventions, including time-skill training and compensation with time-assistive devices to children with ADHD, in addition to medication. This may give better prerequisites for daily life in school-age children with ADHD.

## Electronic supplementary material

Below is the link to the electronic supplementary material.

Supplementary material 1 (DOCX 15 kb)
