# Supplementary material for: Effectiveness of time-related interventions in children with ADHD aged 9–15 years: a randomized controlled study
Source: Eur Child Adolesc Psychiatry. 2017 Sep 27;27(3):329–42. doi: 10.1007/s00787-017-1052-5 (PMC5852175; doi:10.1007/s00787-017-1052-5)
Supplement: Supplementary file 1 — Supplementary material 1 (DOCX 15 kb) [file 787_2017_1052_MOESM1_ESM.docx]

Supplementary data

**Table 3.** P-values for children included in the original and sensitivity analysis with Ancova .

|  | Original data (see table 2) (*n*= 38) | Missing data imputed assuming that the difference between intake and 24-week follow-up would be 0 for the Dropouts (*n*= 46) |
| --- | --- | --- |
| **TPA** | P | p |
| **TPA** |  |  |
| **KaTid - *Sum*** | .019 | .047 |
| **Ka Tid – *Time perception*** | .046 | .043 |
| **KaTid – *Time orientation*** | .010 | .034 |
| **KaTid – *Time management*** | *ns* (.764) | *ns* (.859) |
| **DTM** |  |  |
| **Time - Parent scale** | .011 | .019 |
| **Time - Self-rating** | *ns* (.117) | *ns* (.177) |
